# Brittle Asthma: Still on Board?

**DOI:** 10.3390/biomedicines11113086

**Published:** 2023-11-17

**Authors:** Dina Visca, Francesco Ardesi, Rosella Centis, Patrizia Pignatti, Antonio Spanevello

**Affiliations:** 1 Division of Pulmonary Rehabilitation, Istituti Clinici Scientifici Maugeri, IRCCS, 21049 Tradate, Italy; dina.visca@icsmaugeri.it (D.V.);; 2 Department of Medicine and Surgery, University of Insubria, 2100 Varese, Italy; 3 Clinical Epidemiology of Respiratory Diseases Service, Istituti Clinici Scientifici Maugeri, IRCCS, 21049 Tradate, Italy; 4 Allergy and Immunology Unit, Istituti Clinici Scientifici Maugeri, IRCCS, 27100 Pavia, Italy

**Keywords:** asthma, brittle asthma, unstable asthma, severe asthma, near-fatal asthma

## Abstract

(1) Background: “Brittle Asthma” was considered an asthma clinical phenotype and deemed to be life-threatening in the early 2000s; then, this definition disappeared. The purpose of this review is to examine what has historically been referred to as this term and see whether it may be applied to modern clinical practice, thus acquiring fresh relevance and meaning. (2) Methods: A non-systematic search of the literature was conducted using both MeSH and free-text phrases. No limitations on the research design or type of publication were applied. (3) Results: Reliable data regarding “Brittle Asthma” are lacking due to the paucity of current data and the few studies available. After a few years of reworking, it was divided into two sub-classes: one characterized by a wide PEF variability despite high-dose therapy and the other by sudden acute attacks in otherwise apparently normal airway functions or well-controlled asthma. Their characteristics were hardly defined because of their low prevalence. Data regarding risk factors, atopy, mechanisms, and treatments were analyzed. (4) Conclusions: Over time, different terminology has been introduced to define asthma severity and control. It would be worth investigating whether the term “Brittle Asthma” previously used may be helpful to find new hints to stratify patients and improve disease management.

## 1. Introduction

Bronchial asthma is a chronic inflammatory airway disease resulting from an interaction between environmental and genetic factors. It is characterized by a history of respiratory symptoms varying in intensity and over time, such as wheezing, shortness of breath, chest tightness, and coughing, along with a variable expiratory airflow limitation that can become persistent with disease progression [1]. Bronchial asthma has to be considered as a heterogeneous disease. This definition is due to its variability from an etiological, pathophysiological, clinical, and therapeutic point of view. Based on these characteristics, several classifications have been proposed over time in order to define the so-called “asthma phenotypes” [2,3,4,5]. However, at the moment, these recognizable clusters, based on demographic, clinical, and/or pathophysiological characteristics, have still not been fully investigated and defined [1].

In fact, along with the phenotypic definition, disease severity has also been assessed, entailing a further distinction from the perspective of disease control and therapy. For this purpose, it is essential to explain the terms “control” and “severity”, two distinct entities that may overlap. “Asthma control” is defined as the extent to which the manifestations of asthma have been reduced or removed by treatment. The treatment goal for asthma is to improve respiratory symptoms and to reduce risk factors, such as exacerbations, persistent airflow limitation, and the side effects of therapy. “Asthma severity” is defined as the minimum effective dose of treatment to reach control. Asthma can be stratified for severity according to the treatment needed: mild, moderate, and severe asthma. “Mild asthma” is characterized by disease control obtained with a low-intensity treatment. “Moderate asthma” requires a step three or step four treatment, as suggested by the Global Strategy for Asthma Management and Prevention (GINA) recommendation, to reach control. Asthma is considered “severe” when it requires a high dose of inhaled corticosteroids (plus a second controller) and/or a systemic corticosteroid treatment in order to achieve control, or if it remains “uncontrolled” even after comorbidities are treated [1,6,7,8].

The clinical utility of the term “mild asthma” is debatable, not well defined, and sometimes not recommended to be used because not only moderate and severe but also mild asthma can lead to severe exacerbations [1,9,10]. This clearly underlines the need for new studies on the definition of asthma severity and control. Surely, it will be essential to build on the concept of disease control, and to possibly revive “old” terms and adjust them according to our current knowledge; “Brittle Asthma” is one of those terms on which we would like to focus.

The mortality rate from asthma varies across countries [11,12]. International trends around the 1990s documented a reduction in the death rate [12,13]. However, since the new millennium, epidemiological studies have reported contradictory data. Indeed, in the US, a decrease in mortality has been assessed, while an increased rate in England and Wales has been found [14,15]. “Brittle Asthma” is a clinical phenotype that was considered life-threatening around 2000, being one of the entities at the end of the spectrum severity [16,17]. After the first years of the new millennium, the use of this definition became “silent” and appeared only occasionally in the literature. The GINA document only dedicated a brief paragraph to it, and for the last time, in 2005 [18].

The aim of this review is to go through the concept of “Brittle Asthma” and evaluate whether it could be of any relevance in clinical practice today.

## 2. Materials and Methods

### 2.1. Search Strategy

In order to collect all significative data on the subject, a non-systematic search of the scientific literature in English was carried out without time restrictions. PubMed, EMBASE, Web of Science, and Cochrane Library databases were consulted. A combination of MeSH and free-text terms were used for the search. The following keywords were used: “Brittle Asthma”, “Unstable Asthma”, and “Near-Fatal Asthma”. No limitations on the research or type of publication were applied throughout the literature search. A second analysis of the grey literature has also been conducted to cover every spectrum of the disease entity and every possible reference to it.

### 2.2. Analyses of the Literature

The definition of “Brittle Asthma” was used mainly, even if not as commonly, in recent decades of the twentieth century. In fact, this entity went off the radar after the first few years of the new millennium, with only sporadic appearances in literature.

Due to the lack of current data regarding “Brittle Asthma”, recent information about this condition is absent. Moreover, even in the past, few studies have addressed this issue, and a few trials were carried out to analyze this sub-group of asthma. Because of that, not only are recent data unavailable, but also solid data from the past are lacking, and sometimes only anecdotal data can be found and discussed.

## 3. Definition

In 1977, Turner-Warwick coined the term “Brittle Asthma” to describe individuals with erratic and chaotic wide fluctuations in their peak expiratory flow (PEF), which results in severe asthma attacks that are difficult to manage with standard therapy [19].

About 10 years later, at the end of the 1980s, the criteria to define “Brittle Asthma” were revised in light of daily PEF monitoring. In fact, the definition became more detailed, taking into consideration a diurnal PEF variability greater than 50% on at least three days each week despite the highest level of medical treatment, including high doses of inhaled or oral corticosteroids [20,21].

However, this definition could not yet be considered complete and presented several challenges: the term “maximal therapy” was not defined; the evaluation of “PEF meter readings” was questionable because of poor accuracy of PEF monitoring [22,23]; patients who frequently experienced sudden, severe, and life-threatening attacks despite apparently good asthma control were not included in the definition of “Brittle Asthma” [17,24].

**Figure 1:**
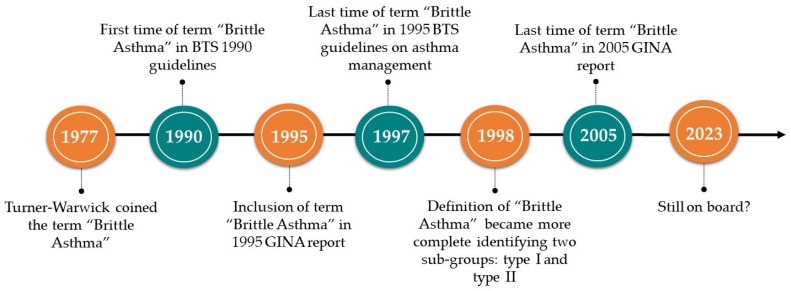
Brittle Asthma timeline [11,12,20,21,23,24,25]. BTS: British Thoracic Guidelines; GINA: Global Initiative for Asthma.

In the 1990s, the British Thoracic Society statement suggested that asthmatic patients recently admitted to the emergency department, or who experienced asthma attacks that became severe (brittle) in a few minutes or hours, should be referred to specialist clinics and followed up for long periods, underlining the importance of labeling uncontrolled or poor controlled asthmatic patients with a red flag [25,26,27,28]. In 1995, the first GINA report defined “Brittle Asthma” as a condition characterized by “highly responsive airways and extreme day-to-day variability in airway obstruction with a risk of sudden, severe and life-threatening exacerbations” [29]. The term “Brittle Asthma” was included in GINA reports until 2005 [18] (Figure 1).

In the 2000s, the definition became more complete and complex. The dose of corticosteroids was specified and the group of asthmatic patients suffering from sudden and unpredictable attacks was included. Therefore, two sub-groups of this phenotype were settled: type I and type II.

The type I brittle asthma sub-class was characterized by a maintained, wide PEF variability (>40% diurnal variation for >50% of the time over a period of at least 150 days) despite considerable medical therapy including a dose of inhaled steroids of at least 1500 μg of beclomethasone or equivalent.

The type II brittle asthma sub-class was characterized by sudden acute attacks occurring in less than three hours without an obvious trigger, on a background of apparent normal airway function or well-controlled asthma [16,24,30].

## 4. Epidemiology

### 4.1. Prevalence

Recent and consistent data about the epidemiology of brittle asthma are absent, and little is known about the incidence and prevalence of this condition. However, most of the articles reported this condition as rare and uncommon [16,31].

Some articles extrapolated data on “Brittle asthma” prevalence from the UK West Midlands Brittle Asthma Register. According to this register, the prevalence could be estimated as around 0.05% of all asthmatic patients. In fact, in a community of approximately 300,000 people affected by asthma, 76 were found to be affected by type I or type II “Brittle Asthma”, and it was estimated that a total of 150 cases were likely to have “Brittle Asthma” [16,30,32,33].

Data regarding gender prevalence seem to be even fewer and not clear. The majority of type I patients are female, with a ratio of 2.5 to 1 male (2.5 F:1 M) and between 18 and 55 years old. Although the reason is unknown, this may be partially due to the development of asthma symptoms in relation to the menstrual cycle [34]. By contrast, patients with type II “Brittle Asthma” seem equally distributed between males and females [16,30,35].

### 4.2. Morbidity, Healthcare Utilization, and Mortality

Clinical aspects that define “Brittle Asthma” recall the concept of asthma severity, associated with a significant burden of morbidity, healthcare utilization, and mortality [30].

The type I sub-group, characterized by patients with prolonged PEF variability, is characterized by a high burden of morbidity and healthcare utilization due to asthma clinical assessment, stabilization, and severe exacerbations. Indeed, it is reported that these patients require longer and more frequent hospitalizations as well as more emergency room visits than type II “Brittle Asthma” patients, whose admissions are shorter and less common, with a more unpredictable rate. In fact, the type II sub-group is characterized by a lower burden of morbidity and healthcare utilization since it is characterized by sudden acute attacks but on a background of apparently normal airway function or well-controlled disease [16,17,30].

Moreover, patients with type I illness are more prone to need high doses of medications, especially corticosteroids, in order to control symptoms and exacerbations, leading to possible iatrogenic side effects. In fact, it is universally known that oral maintenance steroids may cause several side effects such as osteoporosis, glucose intolerance, and weight gain, among others [17,31,33]. Additionally, type I “Brittle Asthma” patients are reported to be more prone to gastro-esophageal reflux (GER). Indeed, even if the link between these two diseases is not completely understood, esophageal reflux was considered as a possible side effect of massive usage of bronchodilators, especially intravenous administration of the nonselective β-agonist, and of theophylline used in the past. Beta-agonists have the ability to reduce the low esophageal sphincter tone and increase GER because they relax smooth muscle [36].

In addition, patients with asthma frequently have hyperinflation leading to increased total lung capacity, residual volume, and functional residual capacity generated by airway obstruction. Esophageal reflux, therefore, may be due to increased negative intra-pleural pressure acting on the lower esophageal sphincter [28].

Type I “Brittle Asthma” has a high morbidity rate, with frequent hospitalizations because of poor disease control and acute or chronic significant drug side effects, including esophageal reflux, osteoporosis, and weight gain related to high use of systemic steroids and inhaled or systemic bronchodilators.

Despite the paucity of data regarding mortality, people with broad fluctuations in PEF are more likely to die from acute asthma; however, an accurate estimation of mortality in types I and II “Brittle Asthma” is unknown [16].

## 5. Risk Factors

Unfortunately, there is a lack of data on risk factors for “Brittle Asthma”, especially for the type II sub-class; to the best of our knowledge, no data have been published on risk factors related to this population. More information about the type I group is available on possible risk factors (i.e. atopy, food intolerance, psychosocial factors, and poor perception of worsening asthma symptoms) [30].

Atopy is defined as the predisposition to synthesize IgE antibodies to allergens and has been considered a well-known risk factor for asthma. In 1995, atopy in “Brittle Asthma” was studied using a skin prick test and Radio Allergo Sorbent Test (RAST), comparing two groups of patients: brittle and non-brittle asthmatics.

At that time, the difference between the type I and type II sub-classes had not yet been assessed; because of that, only one group, which we can consider more similar to type I “Brittle Asthma”, was analyzed.

A prevalence of sensitization to common allergens was found in almost 75.0% of brittle asthmatic patients and in 68.8% of non-brittle asthmatics, without a statistically significant difference between the two groups in terms of prevalence [35].

The majority of patients with type I “Brittle Asthma” were atopic, with at least one positive skin prick test, while a positive reaction to *Dermatophagoides pteronyssinus* was not specific to patients with brittle asthma [30,35,37].

Two third of type I “Brittle Asthma” patients reported at least one “food intolerance”, among which wheat, fish, citrus, eggs, potatoes, soya, peanuts, and yeast were the most common and responsible for poor asthma control [17,30,33]. Indeed, even if the link between “food intolerance” and “Brittle Asthma” has not been studied in depth, it is known that food allergies can cause life-threatening reactions and reduce life quality [38].

Equivalent data for patients with type II “Brittle Asthma” are limited, and exposure to aeroallergens, such as fungal spores, and a poor perception of the disease were considered the main risk factors by several authors [17,30,37].

In one study, it was also documented that total serum IgG and IgA levels were lower in this population [39], suggesting that possible impairment of local immunity could increase the susceptibility to respiratory infections such as viruses associated with more severe asthma [37].

It has been hypothesized that patients with type I “Brittle Asthma” may be significantly affected by psychosocial variables and that emotional factors play a role in the development of the disease. However, it is unclear whether Brittle asthma could cause personality disorders or whether psychological instability may favor the occurrence of “Brittle Asthma”. These individuals frequently struggled to manage their worsening asthma and showed poor compliance to their maintenance therapy, delaying medical visits and either reducing their treatment dose or abusing inhaled or systemic therapy [40,41].

Finally, even when lung function is normal, patients with severe asthma attacks have a decreased perception of worsening airway function and decreased hypoxic drive, suggesting that during an acute attack they may not have a normal ventilatory response, leading to a delay in treatment [30,42,43,44].

In conclusion, risk factors for “Brittle Asthma” patients have not yet been completely identified. However, some clues can be obtained by analyzing risk factors for asthma exacerbations and fatal/near-fatal asthma. Risk factors such as a previous condition of poorly uncontrolled asthma and prior episodes of near-fatal asthma seem to be the strongest predictors. Additionally, other minor factors have been identified such as aeroallergen and smoke exposure, exercise, asthma duration, psychosocial problems, poor medical adherence, old age, aspirin/NSAID sensitivity, numerous asthma hospitalizations, and oral glucocorticoid dependence [1,45,46].

## 6. Mechanisms

Any attempt to elucidate possible mechanisms of “Brittle Asthma” has been hampered by difficulties in performing invasive and non-invasive investigations on these unstable patients. Fiberoptic bronchoscopy and induced sputum would add useful information regarding the inflammatory pattern, the production of cytokines and inflammatory mediators, structural abnormalities, and bronchial innervation of these patients, but their application may be harmful and potentially dangerous. Hypothetical mechanisms may be gathered by studies on severe asthma and fatal/near-fatal asthma.

Acute asthma exacerbation implies a sudden onset of airways narrowing and is less responsive to high doses of β2 agonists, and few data sources available in the literature documented that infiltration of neutrophils rather than eosinophils is typical of a sudden onset attack, along with edema due to plasma exudation from leaky post-capillary venules [17,47].

Smooth muscle contraction of airways is rapidly induced by a cholinergic reflex following exposure to many allergens [30]. Finally, subepithelial collagen and matrix protein disposition, smooth muscle cell hypertrophy, angiogenesis, and goblet cell metaplasia may play a part in the structural alterations of airway remodeling [48].

Several studies, focused on the time of symptoms onset, identified two sub-groups of life-threating asthma attacks: rapid onset (short course) and slow onset (long course) [47,49]. However, the exact time to define the “onset” did not meet a common agreement [50].

The former was defined as an onset of symptoms in less than approximately 3 h, counting around 6–20% of the cases in the majority of the studies [50,51,52,53]. This characteristic is reminiscent of the “Brittle Asthma” definition. In comparison with long course events (approximately more than 6–8 h), which tended to have more mucus in the lumen [49,54], it was discovered that muscle shortening and the ratio of neutrophils to eosinophils were higher in short course attacks, implying a higher concentration of neutrophils [47,49], maybe due to an early detection of airway inflammation [49,55,56,57].

## 7. Treatments

Considering that “Brittle Asthma” is a life-threatening phenotype at the end of the spectrum severity [16], the majority of data and studies on asthma patients highlighted the importance of focusing on preventable and modifiable traits. Non-pharmacological treatment includes the control of allergen exposure, identification and avoidance of allergic food, good dietary support, immunotherapy, and insurance of patient’s compliance. Prevention of asthma genesis, symptoms, and exacerbations could be achieved by avoiding or reducing exposure to risk and trigger factors such as allergens, viral infections, environmental pollutants, tobacco smoke, irritants in general, and medications [1].

By definition, type I “Brittle Asthma” is a form of asthma that occurs with high doses of inhaled corticosteroids. The most common additional treatment was to add systemic steroids and/or large doses of inhaled and systemic β2 agonists, including the long-term subcutaneous infusion of terbutaline, which are all well-known to be responsible for severe acute and chronic side effects. In the case of dependence on steroids, immunosuppressant treatment, such as cyclosporine and methotrexate, was also taken into consideration, acting as steroid sparing [58,59] or intravenous immunoglobulin [60]. However, due to the paucity of data, controversial results, and adverse events, guidelines never recommended the use of these drugs for the treatment of asthma outside of controlled clinical trials.

Patients with type II “Brittle Asthma” are relatively symptom-free between attacks; therefore, non-pharmacological treatment is fundamental and includes the control of allergen exposure, identification of triggers, as well as self-management and management of acute attacks [61]. However, these measures are often difficult to apply comprehensively because most asthmatics react to many ubiquitous factors or show resistance to prevent contact and exposure to some allergens, such as animals, as reported in the unpublished data from the Birmingham case–control study of type I “Brittle Asthma” [30].

In addition, psychological factors could act negatively on asthma control, as previously reported, and psychological treatment may modify the course of the disease [62,63].

Non-compliance (or non-adherence) to the inhaled therapy and incorrect inhaler technique have been considered in asthmatic patients and recognized as a possible reason for poor asthma control. In fact, the GINA document also reports that an incorrect inhaler technique can be seen in up to 80% patients as well as suboptimal adherence in up to 75% of asthmatic patients [1].

Currently, the key points listed above are considered red flags that may alter the asthma clinical course and should be brought to medical attention to be deeply investigated in order to confirm a diagnosis of uncontrolled or severe asthma. Incorrect diagnosis and comorbidities can lead to an uncontrolled and difficult to treat disease.

Novel techniques of prevention and breakthrough treatments for severe asthma have been introduced in the last two decades, aiming to reduce exacerbations and dependence on steroids. Among these, biological therapies could also be an option to improve disease control in “Brittle Asthma”. However, the choice of an appropriate biologic could be difficult due to its unclear pathogenic mechanisms and the lack of published studies. To the best of our knowledge, only one report is available on the use of biological treatment in “Brittle Asthma”, which describes two patients treated with omalizumab, showing the efficacy of this treatment in PEF variability and clinical outcomes [64].

Despite therapeutic novelties in asthma management, severe asthma attacks and uncontrolled asthma are still present and may be life-threatening. According to data of the last WHO report, asthma affected an estimated 262 million people and caused 461,000 deaths in 2019 [65]; however, it is not known how many deaths can be accounted for in the “Brittle Asthma” condition.

Data on mortality in asthma population should refer to all patients suffering from fatal and near-fatal asthma [66,67]. Studies should further investigate this field to evaluate the relationship between patients previously defined in the “Brittle Asthma” era and patients with different grades of asthma control in the light of new treatment available.

## 8. “Brittle Asthma” on the Edge

The concept of “Brittle Asthma” almost disappeared in the early 2000s, while other terms regarding the concept of asthma severity have continued to evolve over the years. Indeed, the term slowly fell into disuse.

One reason that might justify the disappearance of type I “Brittle Asthma” is the need for prolonged monitoring of PEFs to confirm the diagnosis. In fact, one of the main problems in asthma management is patient’s compliance to pharmacological treatment [1]; therefore, it may be reasonable to doubt compliance to daily PEF monitoring over 150 days, especially when patients are well-controlled [68].

Moreover, several studies have tested the numerous mechanical peak flow meters (PFM) developed, finding a lack of accuracy in many devices and significant variability between them [68,69,70,71]. However, recent studies have been trying to find new ways to monitor PEF variability, particularly with digital devices [72].

Currently, daily PEF monitoring continues to have a role in asthma diagnosis and management. Indeed, even as a second choice, it can be used as a test for expiratory airflow variability. The GINA statement suggests two models: short-term and long-term PEF monitoring [1]. In addition, PEF registration is also a useful tool for self-monitoring [1,73].

Short-term monitoring is useful for assessing the response to treatment in general asthma patients, the evaluation of triggers, or to establish a baseline for future action plans. Long-term PEF monitoring is recommended by the GINA statement mainly for severe asthmatic patients, or for those with impaired perception of airflow limitation [1,74,75].

Based on this, beyond asthma diagnosis, PEF monitoring could identify patients with excessive lung function variability, suboptimal control of the disease, and that are at risk of exacerbations [76].

Type II “Brittle Asthma” has been defined as a condition characterized by sudden acute attacks occurring in less than three hours without an obvious trigger and with a background of apparently normal airway function or well-controlled asthma. However, the term type II “Brittle Asthma” has fallen out of use because other terms, covering a wider population, have been introduced in clinical practice and clinical trials to define acute asthma attacks leading to some overlap: near-fatal asthma, life-threatening asthma, moderate acute asthma, and acute severe asthma [77] (Table 1).

## 9. Conclusions and Future Directions

“Brittle Asthma” is a sub-type of asthma introduced in 1977, defined as an uncommon form of severe asthma characterized by a chaotic variation in peak expiratory flow, which could cause life-threating acute severe attacks on a background of poorly controlled asthma despite high doses of inhaled steroids.

Nevertheless, it is reasonable to consider the possibility that patients with a fluctuant pattern of PEF are simply non-compliant with treatment, and they can die from severe asthma attacks due to no optimal treatment. Along with the heterogeneous features of asthma, the term “brittle” can also be used for those patients who are subject to sudden attacks despite usually good asthma control [25,26,27]. In the presence of respiratory symptoms, it is worth mentioning the importance of defining the asthma disease with precision beyond the diagnostic flow-chart. A comprehensive evaluation of the patient at the first visit and during follow-up monitoring in a referral center is needed to better phenotype “Brittle asthma” patients and to assess other comorbidities such as vocal cord dysfunction, gastro-esophageal reflux, immunodeficiencies, bronchiectasis, and cystic fibrosis.

Over the past decades, the term “Brittle Asthma” has been disused and only mentioned in few occasions on GINA documents [18]. However, the terms “difficult-to treat”, “uncontrolled”, “severe”, “near fatal asthma”, and “unstable” are still present in clinical practice, and there is now considerable evidence to suggest that they all play an important part in a poor asthma outcome. An updated definition of “Brittle Asthma” could help to identify a specific asthma phenotype with a high risk of morbidity and/or mortality. This may lead to novel treatment approaches based on lung function variability. Current technology such as electronic meters and smartphone apps to measure PEF could incentivize lung function assessment and novel studies in order to improve the characterization of this phenotype.

In conclusion, future studies are needed to explore whether the term “Brittle Asthma” may still be taken on board and whether its application in clinical practice may improve pharmacological and non-pharmacological treatment, thus reducing morbidity and mortality in relation to asthma.

## Figures and Tables

**Table 1:** Levels of “Asthma Attacks” vs. “Brittle Asthma”.

**Levels of “Asthma Attacks”**		
	**Clinical Signs**	**Measurements**
Moderate acute asthma	Increasing symptoms without a severe condition	PEF > 50–75% best or predicted
Acute severe asthma	Any one of the following:	
	Shortness of breath when talking	PEF 33–50% best or predicted Respiratory rate ≥ 25/min Heart rate ≥ 110/min
Life-threatening asthma	Any one of the following in severe asthma:	
	Altered conscious level Exhaustion Arrhythmia Hypotension Cyanosis Silent chest Poor respiratory effort	PEF < 33% best or predicted SpO_2_ < 92% PaO_2_ < 8 kPa PaCO_2_ (4.6–6.0 kPa)
Near-fatal asthma	Increased PaCO_2_ and/or needing mechanical ventilation with increased inflation pressures	
**“Brittle Asthma”**		
	**Clinical Signs**	**Measurements**
Type I	Despite considerable medical therapy including at least 1500 μg beclomethasone inhaled or equivalent	
	Poor symptoms control	PEF diurnal variability > 40% (>50% of the time) for at least 150 days
Type II	Sudden acute attacks in <3 h without an obvious trigger	
	On a background of apparent well-controlled asthma	Or on a background of apparent normal airway function

PEF: peak expiratory flow; SpO_2_: oxygen saturation measured by a pulse oximeter; PaO_2_: partial arterial pressure of oxygen; kPa: kilopascals; PaCO_2_: partial arterial pressure of carbon dioxide.
